# Do mothers prefer helpers or smaller litters? Birth sex ratio and litter size adjustment in cotton-top tamarins (*Saguinus oedipus*)

**DOI:** 10.1002/ece3.1396

**Published:** 2015-01-08

**Authors:** Rebecca A Boulton, Alison W Fletcher

**Affiliations:** 1 Department of Biological Sciences, University of ChesterChester, U.K; 2 School of Biology, University of St AndrewsSt Andrews, U.K

**Keywords:** Helper repayment hypothesis, *Saguinus oedipus*, sex allocation, sex ratio

## Abstract

Sex allocation theory has been a remarkably productive field in behavioral ecology with empirical evidence regularly supporting quantitative theoretical predictions. Across mammals in general and primates in particular, however, support for the various hypotheses has been more equivocal. Population-level sex ratio biases have often been interpreted as supportive, but evidence for small-scale facultative adjustment has rarely been found. The helper repayment (HR) also named the local resource enhancement (LRE) hypothesis predicts that, in cooperatively breeding species, mothers invest more in the sex which assists with rearing future offspring and that this bias will be more pronounced in mothers who require extra assistance (i.e., due to inexperience or a lack of available alloparents). We tested these hypotheses in captive cotton-top tamarins (*Saguinus oedipus*) utilizing the international studbook and birth records obtained through a questionnaire from ISIS-registered institutions. Infant sex, litter size, mother's age, parity, and group composition (presence of nonreproductive subordinate males and females) were determined from these records. The HR hypothesis was supported over the entire population, which was significantly biased toward males (the “helpful” sex). We found little support for helper repayment at the individual level, as primiparous females and those in groups without alloparents did not exhibit more extreme tendencies to produce male infants. Primiparous females were, however, more likely to produce singleton litters. Singleton births were more likely to be male, which suggests that there may be an interaction between litter size adjustment and sex allocation. This may be interpreted as supportive of the HR hypothesis, but alternative explanations at both the proximate and ultimate levels are possible. These possibilities warrant further consideration when attempting to understand the ambiguous results of primate sex ratio studies so far.

## Introduction

Sex allocation (the relative proportion of parental resources invested in sons and daughters) has been a highly productive area of research within behavioral ecology, demonstrating the remarkable precision of and constraints on adaptation (West et al. 2000; West and Sheldon 2002). Empirical evidence has frequently been found to support theoretical predictions in systems where a mechanism for the facultative adjustment of offspring sex is known to exist (West et al. 2000, 2002). Probably, the most notable case is in the haplodiploid insects, where offspring sex is dependent on whether or not a female fertilizes an egg (resulting in a daughter or son, respectively; Charnov 1982). Parasitoid and fig-pollinating wasps, for instance, produce sex ratios very close to those predicted by local mate competition (LMC) which occurs in spatially structured populations and leads to female-biased sex ratios when sons experience fraternal competition over mates (Hamilton 1967).

Fisher's (1930) principle explains the 1:1 birth sex ratio observed in many species (Charnov 1982; West 2009) using an economic metaphor. If, due to a genetic tendency, more animals of one sex are born, individuals of the rarer sex will become more valuable as parental fitness returns will be higher (assuming that the genetic contribution to future generations is equal for male and female offspring at 1:1). The rarer sex will consequently be favoured by natural selection, causing the sex ratio to return to equilibrium (Frank 1990; West 2009). The theory base initiated by Fisher (1930) was extended by Hamilton (1967) who showed that reasons other than frequency-dependent mating success will contribute to parental fitness, for instance in situations of extreme LMC where a female should produce only enough sons to inseminate all of her daughters; doing so would maximize a female's grand-offspring production. More broadly, parents should invest more in producing the sex which provides them with higher fitness returns (Hamilton 1967; Charnov 1982).

The initial insights of Fisher and Hamilton provided the foundation for several hypotheses to explain why fitness returns from each sex differ and subsequently the generation of quantitative predictions as to the optimal sex ratio an organism should produce given various parameters. In their “maternal condition” hypothesis (TWH), Trivers and Willard (1973) predicted that mothers in good condition will produce more sons, endowed with high competitive abilities due to increased maternal resource allocation. Additionally, females in poor condition will bias the sex ratio toward female offspring, which will reproduce regardless of condition (Trivers and Willard 1973).

Local resource competition (LRC; Hamilton 1967; Charnov 1982) occurs when there is sex-biased dispersal, as the philopatric (nondispersing) sex will compete for resources and so, in net fitness terms, will be more costly to produce leading to the prediction of a sex ratio bias toward the dispersing sex. Another key concept in sex allocation theory is local resource enhancement (LRE) which is also known as the “helper repayment” hypothesis. In many cooperatively breeding species, males and females differ in their tendency to assist in the rearing of future offspring. The helper repayment (HR) hypothesis predicts a sex ratio bias toward the more helpful sex, as individuals who invest more in helping increase their parents' net fitness and will effectively “payback” their own rearing costs (Emlen et al. 1986).

Generally, sex allocation studies yield smaller effect sizes for vertebrates, probably due to chromosomal sex determination (sex is determined by whether an individual is homo- or heterogametic for the sex chromosomes) combined with their complex life histories (West et al. 2005). Despite this, population sex ratios across the vertebrates are often biased (Sheldon and West 2004; Silk and Brown 2008), and Thogerson et al. (2013) showed clearly the adaptive value of sex allocation in terms of grand-offspring production in the mammals. Sex allocation in the primates has yielded a substantial body of literature, and the predictions differ with social system and life history. Despite the copious amount of research into sex allocation in the primates, results have often been inconsistent and difficult to interpret. For instance, when dominance is matrilineally inherited, high-ranking mothers are predicted to produce daughters and low-ranking mothers should produce sons (which will disperse to a new group without the constraints of their mother's low rank), but support has been mixed (supportive: Rhesus macaques *(Macaca mulatta,* Meikle et al. 1984; Long-tailed macaques (*M. fascicularis),* van Schaik et al. 1989; Japanese macaques (*M. fuscata),* Aureli et al. 1990; and no trend/female bias: Bonnet macaques (*M. radiata),* Silk et al. 1981; Savannah baboons (*Papio cynocephalus),* Altmann et al. 1988). Furthermore, a meta-analysis by Brown and Silk (2002) suggested that in studies which consider sex allocation according to dominance rank in the primates, effect size (skewness of sex ratio) decreases in larger samples, implying that the observed trends may be caused by stochastic variation in small samples. In a later meta-analysis, Silk and Brown (2008) suggested that local resource competition (LRC) and helper repayment (HR) played more of a role than maternal condition (TWH) in population sex ratio biases seen regularly in the primates. In cooperatively breeding primates, there is a significant population bias toward males (such as in callitrichids), where males are typically better helpers than females, through increased investment in infant carrying, food provisioning (Dunbar 1995), and later dispersal, providing help in the natal environment for longer (McGrew and McLuckie 1986). However, Pen and Weissing (2000) argue that if parents are facultatively adjusting the sex of their offspring according to the benefit of having helpers, the population sex ratio will not necessarily be biased toward the helpful sex. Often, systems with sex-biased cooperative breeding will also have sex-biased dispersal, so any effect of adjusting offspring sex according to the helper repayment hypothesis (HR) may be countered by local resource competition (LRC, Koenig and Walters 1999; Pen and Weissing 2000). In the Seychelles warbler (Komdeur 1992, 1996; Komdeur et al. 1997), for example, helpers are only beneficial on good quality territories; on poor territories, it is better to produce an unhelpful, but dispersive female to alleviate resource competition. In early tests of helper repayment, Gowaty and Lennartz (1985) did find a significant bias toward the helpful sex (males) in the cooperatively breeding red-cockaded woodpecker (59% males), but Walters (1990) did not (49% males); a population-level sex ratio bias alone does not unequivocally support or refute the HR hypothesis.

Koenig and Walters (1999) suggest investigation into the individual level effects of certain factors, in accordance with the predictions of the helper repayment hypothesis, in order to facilitate greater understanding of these processes across the vertebrates. For instance, in cooperatively breeding mammals such as alpine marmots (*Marmota marmota;* Allainé 2004) and African wild dogs (*Lyacon pictus*; Griffin et al. 2005), birth sex ratios are more biased toward the helpful sex in young, primiparous mothers than in older, multiparous mothers. This is adaptive because a young female who has recently dispersed from her natal group must establish her own group, which may only consist of herself and her mate. Producing more of the helpful sex early on will be beneficial in terms of future reproduction, as a mother can benefit most from their help in rearing the subsequent litter. Further support for this possibility comes from the cooperatively breeding carrion crow (*Corvus corone corone)*. In this species, males are the helpful sex, and Canestrari et al. (2012) found that in groups with few subordinate male helpers, more male chicks were fledged.

Although Silk and Brown (2008) found support for the helper repayment hypothesis in primates, their meta-analysis only considered population sex ratio biases. Recently, Rapaport et al (2013), Kloc, Warneke, Mickelberg, and Ballou (2013) considered how factors such as mother's age and group size might affect sex allocation strategies at the individual level in two cooperatively breeding captive primate species, golden lion tamarins (*Leontopithecus rosalia*) and callimicos (*Callimico goeldii*) which typically produce twin and singleton litters, respectively. Although they showed that group size does influence offspring survival, they did not find any adaptive response in terms of sex allocation within species. Young and first time mothers were no more likely to produce sons, and there was no effect of group size on male offspring production.

The aim of the current study was, such as Rapaport et al. (2013), to test the predictions of the HR hypothesis at the individual level in a cooperatively breeding callitrichid, the cotton-top tamarin (*Saguinus oedipus*). Like *C. goeldii* and *L. rosalia*, reproductive success in *S. oedipus* is constrained largely by the presence of helpers (males in particular) to assist in offspring rearing (Bardi et al. 2001). Despite a lack of support for the HR hypothesis, Rapaport et al. (2013) did find that in the twinning species, *L. rosalia*, the sex ratio in singleton births was 65% male compared to 55% in twin litters. They suggest the population-level male bias results from in utero litter size reduction which is instigated by male fetuses (at the expense of female litter mates). If males are competitively superior, then this may mask any facultative control the mother exerts. Unlike *C. goeldii* and *L. rosalia*, which typically produce singleton and twin offspring, respectively, *S. oedipus* females produce litters ranging in size from 1 to 4 (although 69% are dizygotic twins). An additional aim of the current study was to test whether litter size modification might interact with birth sex ratio, for instance, if, as Rapaport et al. (2013) propose, there is sex-biased reabsorption of fetuses or if one fetus is competitively superior in utero (and this competitive superiority is also sex-biased; Jaquish et al. 1996; Tardif and Jaquish 1994; Rapaport et al. 2013). We will also investigate whether females vary their litter size depending on environmental and life-history variables (such as parity, age, and group size) which provide reliable information on likely infant survival (Tardif et al. 2013 ).

## Methods

Data were compiled using an electronic questionnaire which was sent by e-mail to all ISIS (2008)-registered (International Species Information System) institutions housing cotton-top tamarins (*Saguinus oedipus)* requesting details of the sex of any infants born, litter size and dates of all births, deaths and transfers as well as the mother's date of birth and parity (which were contained in their ARKS; Animal Record Keeping System database). Additionally, the international studbook keeper and EEP (European endangered species breeding programmes) coordinator were approached in order to gain access to the Single Population Analysis and Record Keeping System (SPARKS) records for *S. oedipus*. The international studbook for the cotton-top tamarin was established in 1986, but only data from a 10-year period (01/13/1999–07/25/2009) were included in this study as many earlier entries were from wild caught animals whose age and parity could not be reliably determined.

Age of the mother (in months, at the estimated time of conception, 6 months prior to parturition, French et al. 2002) and parity (primiparous or multiparous) as well as litter size were then determined using the ARKS data provided by institutions and the birth records listed in the SPARKS database. Additionally, the ARKS data (on births, deaths, and transfers) were used to determine the total group size and composition (number of nonreproductive males and nonreproductive females) present at the time of conception.

### Statistical analysis

Once data sets were combined and all unsexed births and duplicated data points had been removed, a total of 1784 infants (from 1146 litters and 387 mothers in 208 zoos) were available for statistical analysis of maternal age, parity, and litter size; this included 267 singletons (15%), 1235 twins (69.2%), 270 triplets (15.1%), and 12 quadruplets (0.7%). These births represented data extracted from the SPARKS database and combined with ARKS records provided by individual institutions. Forty-eight institutions also provided information regarding group size and composition; these data were available for 576 infants (representing 294 litters from 90 mothers).

While Rapaport et al. (2013) excluded infants who were hand-reared, rearing status was unavailable for *Saguinus oedipus,* and we considered the sex ratio of litters where all infants were born alive and the sex was determined.

Generalized linear mixed models (GLMMs using lme4 in R) were used to determine whether any of the aforementioned variables influenced the probability of giving birth to a male. Binary logistic regression with a binomial error structure (0 = female, 1 = male) and a logit link function were used (after calculating dispersion parameters which indicated the data were not overdispersed) to determine the effects of the independent variables on the binary (dichotomous) dependent variable (sex). Independent variables were mother's age in months (entered as a covariate) and parity (a fixed factor; primiparous or multiparous). Litter size (1–4) was also entered as a fixed factor; additional fixed factors included alloparent presence (whether there were nonreproductive subordinate (NRS) animals of either sex in the group at the time of conception) and the sex of NRSs (i.e., the number of nonreproductive males or females were considered separately). Zoo population, mothers' identity, and litter identity were entered as nested random factors in the model as infants from the same population, mother, and litter cannot be treated as independent observations (Bolker et al. 2009; Krackow and Tkadlec 2001). An additional GLMM included the same random and predictor variables (mother's age, parity, and alloparent presence and sex) with litter size as the outcome variable (quasi-Poisson error structure, which is robust to over and underdispersion) to determine whether females appear to modify their litter size in response to social or life-history variables.

## Results

### Sex ratio

**Figure 1:**
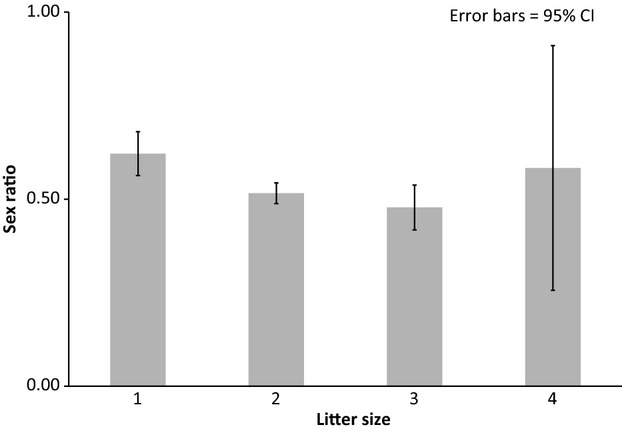
Variation of sex ratio (proportion males) with litter size. Infants in single litters are more likely to be male than in triplet litters.

Birth sex ratio was determined by the proportion of males born (BSR_M_). From complete litters in the entire sample, the BSR_M_ was 0.53; this male bias was shown to be significantly different from the expected 0.5 (1:1) (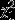 = 4.72, *P *=* *0.03). Singleton births were more likely to be male than litters containing multiple infants (Fig.1). The sex ratio was significantly more male-biased in singleton births than litters containing multiple infants (Table1). No variables other than litter size significantly predicted infant sex (Table1). When the model included the complete data set (ARKS and SPARKS; which did not include group size or composition, see supplementary material) the results did not change; litter size remained the only significant predictor of infant sex.

**Table 1:** Summary of GLMM examining the effect of litter size, mother's age, parity, and NRS presence & sex on offspring sex ratio (df = 3 for litter size, df = 1 for all other predictors)

	B estimate ± SE	*χ* ^2^	*P*
Intercept	0.13 (0.30)		
Litter size		6.42	0.04
Single versus twin	−0.30 (0.28)		0.28
Single versus triplet	−0.76 (0.33)		0.02
Twin versus triplet	−0.59 (0.34)		0.08
Parity	−0.18 (0.34)	0.10	0.74
Mother's age	−0.00 (0.00)	0.16	0.69
All NRSs	0.49 (0.59)	0.45	0.50
Male NRSs	−0.36 (0.44)	0.69	0.41
Female NRSs	−0.02 (0.34)	0.00	0.95

Removing litter size from the model did not suggest that any other variables (which may be correlated with litter size) were related to the birth sex ratio. Furthermore, an additional model considered only twin births in order to determine whether life-history variables and group size/composition have any effect on the sex ratio independently of litter size, but results did not differ from the original model (see supplementary material).

### Litter size

**Figure 2:**
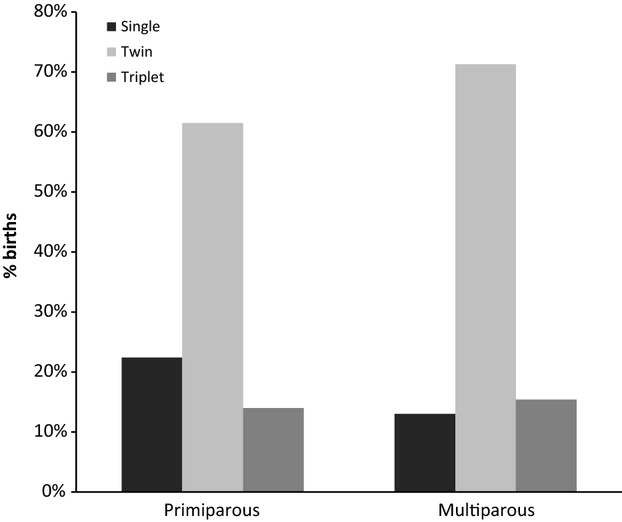
Percentage of infants born as singletons, or in twin or triplet litters by parity.

Fifteen percent of all births were single infants, 69.2% were twins, and 15.1% were triplets. Of the entire sample, only 12 quadruplet litters were born (0.7%). Litter size could be under proximate maternal control, and therefore, an additional GLMM was used to determine whether mother's age, parity, and presence of NRSs had any effect on litter size (Table2). Only parity had a significant effect on litter size; the first (primiparous) birth was more likely to be a single infant than subsequent births (Fig.2). The effect of parity increased, but remained the only significant predictor of litter size when the complete data set (ARKS and SPARKS) was used (see supplementary material).

**Table 2:** Summary of GLMM examining the effects of mother's age, parity, and NRS presence and sex (df = 1 for all predictors) on the litter size

	B estimate ± SE	*χ* ^2^	*P*
Intercept	0.67 (0.05)		
Parity	0.05 (0.03)	0.17	0.05
Mother's age	−0.00 (0.00)	0.01	0.60
All NRSs	−0.03 (0.05)	0.02	0.54
Male NRSs	0.02 (0.04)	0.01	0.71
Female NRSs	0.03 (0.03)	0.06	0.23

## Discussion

The results of the current study indicate that in the captive cotton-top tamarin (*Saguinus oedipus*), the overall birth sex ratio (BSR) was significantly male-biased. On a simple level, this is consistent with the predictions of the helper repayment (HR) hypothesis (Emlen et al. 1986), but despite this consistency, the HR hypothesis may not explain the male bias observed. Pen and Weissing (2000) argue that a population sex ratio bias alone does not unequivocally support or refute the HR hypothesis as environmental conditions might interact to reduce the benefit of producing the helping sex. Furthermore, other factors may contribute to population-level male bias seen here (i.e., Silk and Brown 2008; Rapaport et al. 2013), for instance greater potential fitness returns from sons, who, in the wild at least, have more opportunities for extra-group mating than subordinate daughters who are reproductively suppressed by the dominant female in a group (Ziegler et al. 1987).

In this study, we found little support for the prediction that young and primiparous mothers, or even those with no nonreproductive alloparents, would produce more of the helpful sex (sons) than older or multiparous mothers with helpers. The failure for young/primiparous mothers to increase son production could result from alloparental care which is not fully sexually asymmetric in callitrichids. Subordinate females do assist in infant care, but to a lesser extent than males (Price 1992). If the fitness benefit of producing a son versus a daughter is not sufficient to increase fitness substantially, there would be no expected effect of age, parity, and alloparent presence on birth sex ratio itself. Price (1992) found that juvenile daughters actually provide more care than juvenile sons. Females do not continue this investment into adulthood, suggesting that they use this experience to assist their own reproduction later in life (Sánchez et al. 2002). As such, the sex of the first offspring may not matter enormously, particularly given the low probability of survival of the first infant; in captive colonies, maternal experience explains 16.8% of the variance in infant survival (Bardi et al. 2001).

Although the proximate mechanisms underlying mammalian sex allocation have often been fairly speculative, it seems unlikely that there would be no proximate mechanism for biasing offspring sex in mammals, given its potential adaptive value (Thogerson et al. 2013). A recent study has shown that female pigs can modify the oviductal environment in response to sperm type, suggesting that female mammals are capable of biasing offspring sex through differential local immune responses to X- or Y-bearing spermatozoa (Almiñana et al. 2014). In the callitrichids, female energy status is a key variable influencing reproductive investment (Tardif et al. 2013), with high levels of glucose enhancing male blastocyst development (Navara and Nelson 2009). This could potentially explain both the male bias in captive populations (due to reduced nutritional stress) and why females do not seem to adjust sex allocation according to the variables under investigation here (mother's age, parity, & alloparent presence). If females can use their energetic status as a proxy for current conditions (be they life history, environmental, or social), any ability to allocate sex adaptively may be masked by a lack of variation in nutritional status (van Dooren and Leimar 2003; Schwanz and Proulx 2008). This is consistent with the results of the current study; if this kind of phenotypic plasticity dictates sex allocation decisions according to maternal energy status, then we would expect both a surplus of males in captivity and insufficient variation in maternal condition to see the outcome of sex allocation decisions at the individual level. In captivity, alloparental presence and female age/experience may thus not influence maternal energy status sufficiently (due to the dominant effect of enhanced nutrition) to observe individual level variation in sex allocation decisions.

Although the current study found only circumstantial evidence for helper repayment, we did find evidence to suggest that primiparous females are more likely to produce a single infant. In captivity, litter size may be a more important reproductive outcome than infant sex. This might reflect the fact that the key determinant of reproductive fitness in captivity, where availability of resources is not an issue, is infant mortality due to maternal inexperience (whereas in the wild, the limiting factor for infant survival is resource availability; Snowdon et al. 1985). The rate of stillbirth and subsequent neonatal mortality is particularly high for the first litter a female produces (Leong et al. 2004; Tardif et al. 2013), and in captive callitrichids, infants born in smaller litters are more likely to survive the neonatal period (Jaquish et al. 1991; Leong et al. 2004). A primiparous female should therefore concentrate her efforts on gestating and rearing a single infant in order to improve survival. Investing more resources into a single infant will increase survivorship when mothers are inexperienced. This will result in a higher net fitness gain both directly through care for future offspring by nonreproductive subordinates and indirectly through inclusive fitness. Females may thus potentially benefit by not over-investing (i.e., in multiple infants) in early reproductive attempts where the likelihood of success is low.

In addition to finding that primiparous females were more likely to give birth to a single infant, we also found that the probability of producing a male infant was greater in singleton births. The majority of callitrichid litters are dizygotic (nonidentical) twins and triplets, but litter sizes of up to six have been recorded in captive *Callithrix jacchus* (Tardif et al. 2013). Although litter size is dictated largely by the number of ova matured and released (Tardif and Ross 2009), single infant births typically result from reabsorption of one or more embryos (in the callitrichids, this is facilitated by delayed embryonic development; females have an extended period of time in which to cease investing in a reproductive attempt if it is no longer profitable to do so; Tardif and Ross 2009). This suggests that primiparous females may reabsorb one or more embryos to reduce their current reproductive burden and optimize their future reproductive success (Tardif and Jaquish 1994, 1997; Jaquish et al. 1996). The male bias in singleton births could result from either a maternal preference for reabsorbing female embryos or asymmetric embryonic competitive ability, as suggested by Rapaport et al. (2013). Whether maternal embryo preference or differential competitive ability exists requires investigation, but may be a promising avenue for future research given the potential for altering reproductive investment postinsemination. Regardless of the proximate explanation, producing a male infant early on is expected to help maximize future reproduction if the quality of male alloparental care is higher (McGrew and McLuckie 1986; Dunbar 1995).

There does appear to be an interaction between litter size and sex allocation; these reproductive decisions may not be independent. Many reproductive decisions appear to be related to maternal energy balance or condition as well as cortisol levels (Grant 1996, 2007), which we expect to vary with age and parity (Bales et al. 2005), as well as previous reproductive investment (which will also relate to the amount of alloparental care available). Although the direct effect of parity on sex ratio was not statistically significant, the finding that a primiparous female is more likely to produce a single infant, and that a single infant is more likely to be male does suggest some degree of facultative control over sex after insemination. In a similar way, female carrion crows can maximize male fledgling success by allocating offspring sex ratio along the hatching sequence (the first chicks to hatch have higher survivorship; Canestrari et al. 2012). By doing so, carrion crow mothers can produce more sons in a group which was previously short of (helpful) subordinate males. It is possible that in *Saguinus oedipus* too, a mother producing a single male infant increases this offspring's chances of survival and therefore also increases the success of her future offspring due to increased alloparent availability. Data in the studbook could be further utilized to test this prediction and whether the secondary sex ratio (i.e. infant survival to independence from the mother) is greater for males in first births depending on litter size.

A recent study has highlighted the potential for natural selection to act on the facultative adjustment of birth sex ratio; Thogerson et al. (2013) found, using data from captive breeding programmes for 198 mammalian species, that grandparents which produced sex ratios which were biased in the predicted direction had more grand-offspring. Clearly, primates and other mammals can maximize their fitness returns by allocating sex, even in captivity (Thogerson et al. 2013), but the results of the current study suggest that reproductive decisions are not so unequivocal. Other reproductive decisions, such as how many infants to gestate and support, may take initial precedence in terms of maximizing fitness returns. These decisions may, however, interact with the primary or secondary sex ratio, perhaps due to maternal preference or asymmetric pre- and postnatal competition between the sexes. The callitrichid primates represent a valuable study system for further investigation and modeling of mammalian reproductive decisions in the wild and captivity.

## Supporting Information

**Table S1.**Summary of GLMM (binomial error distribution, dispersion parameter = 1.00) examining the effect of litter size, parity and mother's age on offspring sex ratio (df = 3 for litter size, df = 1 for all other predictors) which included the complete SPARKS & ARKS dataset (*N* = 1743 infants). **Table S2.** Summary of GLMM (binomial error distribution, dispersion parameter = 1.01) examining the effect of parity and mother's age on offspring sex ratio (df = 3 for litter size, df = 1 for all other predictors) which included the ARKS dataset (*N* = 373 infants). **Table S3.** Summary of GLMM (binomial error distribution, dispersion parameter = 1.00) examining the effect of parity and mother's age on offspring sex ratio (df = 3 for litter size, df = 1 for all other predictors) which included the ARKS dataset (*N* = 1274 infants). **Table S4.** Summary of GLMM (binomial error distribution, dispersion parameter = 1.03) examining the effect of parity, mother's age, alloparent presence and sex on offspring sex ratio (df = 1 for all predictors) which included only twin births from the ARKS dataset (*N* = 207 infants). **Table S5.** Summary of GLMM (binomial error distribution, dispersion parameter = 1.00) examining the effect of parity and mother's age on offspring sex ratio (df = 1 for all predictors) which included only twin births from the SPARKS & ARKS dataset (*N* = 1274 infants). **Table S6.** Summary of GLMM (quasipoisson error distribution, dispersion parameter = 0.16) examining the effect of mother's age and parity on litter size (df = 1 for all predictors) which included the complete SPARKS & ARKS dataset (*N* = 972 litters).
