# Supplementary material for: Do mothers prefer helpers or smaller litters? Birth sex ratio and litter size adjustment in cotton-top tamarins (Saguinus oedipus)
Source: Ecol Evol. 2015 Jan 8;5(3):598–606. doi: 10.1002/ece3.1396 (PMC4328765; doi:10.1002/ece3.1396)
Supplement: Supplementary file 1 [file ece30005-0598-sd1.docx]

***SUPPLEMENTARY MATERIAL***

**Table S1. Summary of GLMM (binomial error distribution, dispersion parameter = 1.00) examining the effect of litter size, parity and mother’s age on offspring sex ratio (df = 3 for litter size, df = 1 for all other predictors) which included the complete SPARKS & ARKS dataset (N = 1743 infants)**

|  | *B estimate ± SE* | | *X^2^* | *P* |
| --- | --- | --- | --- | --- |
| Intercept | 0.47 (0.24) |  |  |  |
| Litter size |  |  | 12.19 | 0.007 |
| Single vs twin | -0.49 (0.13) |  |  | 0.004 |
| Single vs triplet | -0.38 (0.19) |  |  | 0.04 |
| Single vs quad | -0.51 (1.01) |  |  | 0.61 |
| Twin vs triplet | 0.11 (0.15) |  |  | 0.48 |
| Twin vs quad | -0.02 (1.00) |  |  | 0.98 |
| Triplet vs quad | -0.13 (1.02) |  |  | 0.90 |
| Parity | 0.11 (0.13) |  | 0.72 | 0.39 |
| Mother’s age | -0.00 (0.00) |  | 1.03 | 0.30 |

**Table S2. Summary of GLMM (binomial error distribution, dispersion parameter = 1.01) examining the effect of parity and mother’s age on offspring sex ratio (df = 3 for litter size, df = 1 for all other predictors) which included the ARKS dataset (N = 373 infants)**

|  | *B estimate ± SE* | | *X^2^* | *P* |
| --- | --- | --- | --- | --- |
| Intercept | 0.67 (0.55) |  |  |  |
| Parity | -0.35 (0.33) |  | 0.85 | 0.36 |
| Mother’s age | 0.00 (0.00) |  | 0.08 | 0.78 |
| All NRSs | 0.60 (0.58) |  | 0.29 | 0.59 |
| Male NRSs | -0.42 (0.44) |  | 0.90 | 0.34 |
| Female NRSs | 0.07 (0.33) |  | 0.05 | 0.82 |

**Table S3. Summary of GLMM (binomial error distribution, dispersion parameter = 1.00) examining the effect of parity and mother’s age on offspring sex ratio (df = 3 for litter size, df = 1 for all other predictors) which included the ARKS dataset (N = 1274 infants)**

|  | *B estimate ± SE* | | *X^2^* | *P* |
| --- | --- | --- | --- | --- |
| Intercept | 0.13 (0.22) |  |  |  |
| Parity | 0.05 (0.13) |  | 0.19 | 0.66 |
| Mother’s age | -0.00 (0.00) |  | 0.92 | 0.34 |

**Table S4. Summary of GLMM (binomial error distribution, dispersion parameter = 1.03) examining the effect of parity, mother’s age, alloparent presence and sex on offspring sex ratio (df = 1 for all predictors) which included only twin births from the ARKS dataset (N = 207 infants)**

|  | *B estimate ± SE* | | *X^2^* | *P* |
| --- | --- | --- | --- | --- |
| Intercept | 1.23 (0.73) |  |  |  |
| Parity | -0.72 (0.12) |  | 2.44 | 0.12 |
| Mother’s age | -0.00 (0.00) |  | 0.00 | 0.98 |
| All NRSs | 0.27 (0.74) |  | 0.20 | 0.66 |
| Male NRSs | -0.26 (0.56) |  | 0.29 | 0.59 |
| Female NRSs | 0.16 (0.43) |  | 0.15 | 0.69 |

**Table S5. Summary of GLMM (binomial error distribution, dispersion parameter = 1.00) examining the effect of parity and mother’s age on offspring sex ratio (df = 1 for all predictors) which included only twin births from the SPARKS & ARKS dataset (N = 1274 infants).**

|  | *B estimate ± SE* | | *X^2^* | *P* |
| --- | --- | --- | --- | --- |
| Intercept | 0.16 (0.27) |  |  |  |
| Parity | 0.02 (0.15) |  | 0.02 | 0.19 |
| Mother’s age | -0.00 (0.00) |  | 1.72 | 0.87 |

**Table S6. Summary of GLMM (quasipoisson error distribution, dispersion parameter = 0.16) examining the effect of mother’s age and parity on litter size (df = 1 for all predictors) which included the complete SPARKS & ARKS dataset (N = 972 litters)**

|  | *B estimate ± SE* | | *X^2^* | *P* |
| --- | --- | --- | --- | --- |
| Intercept | 0.46 (0.04) |  |  |  |
| Parity | 0.09 (0.02) |  | 2.97 | 0.0007 |
| Mother’s age | -0.00 (0.00) |  | 0.00 | 0.85 |
